# Genetic aberrations in Chinese pancreatic cancer patients and their association with anatomic location and disease outcomes

**DOI:** 10.1002/cam4.3679

**Published:** 2020-12-22

**Authors:** Junliang Lu, Ruoying Yu, Rui Liu, Xiaolong Liang, Jian Sun, Hui Zhang, Huanwen Wu, Zhiwen Zhang, Yang W. Shao, Junchao Guo, Zhiyong Liang

**Affiliations:** ^1^ Department of Pathology Molecular Pathology Research Center Peking Union Medical College Hospital Chinese Academy of Medical Sciences & Peking Union Medical College Beijing China; ^2^ Translational Medicine Research Institute Geneseeq Technology Inc Toronto ON Canada; ^3^ Department of Pathology Beijing Chaoyang Hospital Capital Medical University Beijing China; ^4^ Nanjing Geneseeq Technology Inc Nanjing Canada; ^5^ School of Public Health Nanjing Medical University Nanjing China; ^6^ Department of General Surgery Peking Union Medical College Hospital Chinese Academy of Medical Sciences & Peking Union Medical College Beijing China

**Keywords:** body and tail of pancreas, disease‐free survival, DNA Damage Repair pathway, head and neck of pancreas, KRAS subtype, overall survival, pancreatic cancer

## Abstract

**Objectives:**

Pancreatic cancer (PC) is one of the most lethal malignancies with an increasing death rate over the years. We performed targeted sequencing and survival analyses on 90 Chinese pancreatic cancer patients, hoping to identify genomic biomarkers associated with clinical outcomes and therapeutic options.

**Method:**

Genomic DNA was extracted from formalin‐fixed paraffin‐embedded (FFPE) tissue specimens of 90 pancreatic cancer patients and sequenced. The associations with clinicopathological factors were analyzed.

**Result:**

High prevalence of driver mutations in *KRAS*, *TP53*, *CDKN2A*, *SMAD4*, and *ARID1A* genes were found. Most mutated genes in PC belonged to cell cycle and DNA damage repair pathways. Tumors that arise from the pancreas’ body and tail (BT tumors) displayed a higher ratio of mutated *KRAS* and *TP53* than those that arise from the pancreas’ head and neck (HN tumors), who showed less diverse *KRAS* subtypes. Patients with a *KRAS* p.G12R mutated tumor tended to have a prolonged disease‐free survival (DFS) and overall survival (OS) than other *KRAS* subtypes. Those with an altered *ARID1A* gene and more than two mutated driver genes tended to have a shorter DFS and OS.

**Conclusion:**

HN and BT tumors of the pancreas displayed different mutational profiles, which had prognostic significances and indicated different potential therapeutic options.

## Graphical abstract

We analyzed the germline and somatic mutations on 90 Chinese pancreatic cancer patients. We found that a large proportion of Chinese pancreatic cancer patients harbored mutations of the DNA damage repair pathway genes, and that adenocarcinomas of the pancreatic head and neck showed different mutation profile to those of the pancreaitc body and tail. Our finding might provide provision to potential therapuetic options and facilitate future clinical trial allocations

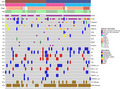

## 1 INTRODUCTION

Pancreatic cancer (PC) is a malignancy that confers a high mortality rate.^1^ The death toll due to PC is increasing over the years, and PC is predicted to be the second leading cause of cancer‐related death by 2030.^2^ New strategies for late‐stage PC treatment are being evaluated, including new chemotherapeutic regimens, immunotherapies, and small‐molecular inhibitors that target the oncogenic pathways, emphasizing the need to improve the understanding of cancer genome and transcriptome alterations. Current data produced by massive parallel sequencing is overwhelming. They either identify crucial driver genes of the disease or subclassified PC into different molecular categories associated with patient survival.^3^

Molecular testings have been adopted to inform the optimal therapeutic strategies for many cancers. However, in pancreatic cancer, only germline *BRCA1/2* and *PALB2* mutation, microsatellite instability, and NTRK fusions have gained general acceptance as actionable alterations by experts in the field.^4^, ^5^, ^6^

Two years have passed since the publication of Pishvaian et al. on the molecular profile of 640 pancreatic cancer cases, where they reported that 50% of the patients harbored actionable genomic alterations.^7^ Given the rapid accumulation of knowledge on novel targets and the presence of new therapeutic compounds, some previously undruggable mutations might now become targeted. Thereby, we assume that it might still be informative to probe the known cancer‐related genes in a Chinese pancreatic cancer cohort to explore the clinicopathological significance and identify possibly druggable cases according to their mutational profile.

## 2 MATERIALS AND METHODS

### 2.1 Patients and sample collection

The research was approved by the Institutional Review Board of Peking Union Medical College Hospital. Written informed consent to participate was obtained from each patient. The flow chart of patient enrollment was shown in Figure S1. Totally 90 pancreatic cancer patients were recruited via the pathology department's electronic record, and experienced pathologists reviewed the Hematoxylin‐Eosin (H&E) stained slides. Representative formalin‐fixed paraffin‐embedded (FFPE) tissue blocks containing cancer regions and non‐cancer regions were retrieved from the pathology archives of the department. Resected specimens were chosen. Five‐micrometer‐thick FFPE sections made from the representative tissue blocks were then submitted for analyses by a 425‐gene target‐capture next‐generation sequencing panel.

### 2.2 Clinical data

Demographic and clinical information, including sex, age, clinical presentation, resectability, evidence of distant metastasis, and relevant family histories, were retrieved from the hospital information system. Of note, tumors located to the right of the superior mesenteric vein (SMV) was considered to have arisen from the pancreas’ head and neck (HN), while that located to the left of the SMV was considered to have arisen from the pancreas’ body and tail (BT). Follow‐ups were conducted via either telephone or in the clinic. Patients were excluded from clinicopathological analyses if they met any of the following criteria^1^: sample with no detectable mutations or low quality,^2^ sample without tumor primary site information, and^3^ sample without disease‐free survival (DFS) and overall survival (OS) information.

### 2.3 DNA extraction and quantification, library preparation

FFPE samples were de‐paraffinized with xylene, and DNA was extracted using the QIAamp DNA FFPE Tissue Kit (Qiagen) according to the manufacturer's protocols. Purified DNA was qualified by Nanodrop2000 (Thermo Fisher Scientific) and quantified by Qubit 2.0 using the dsDNA HS Assay Kit (Life Technologies) according to the manufacturer's recommendations. Sequencing libraries were prepared using the KAPA Hyper Prep kit (KAPA Biosystems) with an optimized manufacturer's protocol and sequenced as previously described.^8^ The tumor was sequenced to an average depth of 600–700X, while corresponding normal tissue was sequenced to at least 30X of depth on an Illumina Hiseq 4000 platform (Illumina).

### 2.4 Data processing

Sequencing data were processed as previously described.^9^ In brief, the data were first demultiplexed and subjected to FASTQ file quality control to remove low‐quality data or N bases. Qualified reads were mapped to the reference human genome hg19 using Burrows–Wheeler Aligner and Genome Analysis Toolkit (GATK 3.4.0) was employed to apply the local realignment around indels and base quality score recalibration. Picard was used to remove PCR duplicates. VarScan2 was employed for the detection of single‐nucleotide variations (SNVs) and insertion/deletion mutations. ADTEx was used to identify copy number variations (CNVs) with a reference human DNA sample NA18535 as the control. The cut‐off of log2 ratio was set at ±0.6 for copy number changes (corresponding to 1.5‐fold copy number gain and 0.65‐fold copy number loss). The classification of germline mutations was based on the guidelines of the American College of Medical Genetics and Genomics and the Association for Molecular Pathology (referred to as the ACMG guidelines thereafter), where Class 4 and 5 mutations were referred to as deleterious mutations thereafter.^10^ Somatic mutations and therapeutic implications were annotated according to the OncoKB database, where Tier I and II mutations were referred to as actionable and potentially actionable mutations, respectively, in the following text.^11^

### 2.5 Statistics

The associations between gene alterations (mutations, CNV, chromosome gain, and loss) and clinicopathological factors were tested using Fisher's exact test. Fisher's exact test was used for pathway analysis as well. The correlation of gene alterations and tumor locations were tested using Fisher's exact test. Survival analyses, including DFS and OS, were performed using the Kaplan–Meier method. Univariate and multivariate analyses, if appropriate, were conducted using the Cox proportional hazards model.

## 3 RESULTS

### 3.1 Patient overview

The clinicopathological characteristics of 90 pancreatic cancer patients were presented in Table 1. The median age of patients was 59 years, ranging from 36 to 80 years. The majority of the patients had pancreatic ductal adenocarcinomas (PDAC, 92.2%, 83 out of 90). The cohort comprised slightly more males (55.6%, 50 out of 90) than females (44.4%, 40 out of 90). Most of the patients were at stage 2 upon diagnosis (83.3%, 75 out of 90). Regarding the primary tumor location, the number of patients with BT cancers was equivalent to those with HN cancers. About 42.2% (38 out of 90) of pancreatic cancer patients present with gastrointestinal symptoms. All cases were resectable or borderline resectable when assessed preoperatively, according to the consensus statement of Abdominal Radiology and the American Pancreatic Association.^12^ All but three cases (3.33%) had macroscopic residual tumor after surgery.

**TABLE 1:** Demographic characteristics and survivals of 90 pancreatic cancer patients

Feature	Categories	Count	Column N%	Overall survival (median)	Log‐rank p	Progression‐free survival (median)	Log‐rank *P*
Age (years)	<=65	67	74.4%	27.0	0.632	18.0	0.907
	>65	23	25.6%	24.0		24.0	
Sex	Male	50	55.6%	27.0	0.282	18.0	0.173
	Female	40	44.4%	31.0		16.0	
Histological subtype	Ductal	83	92.2%	27.0	0.428	18.0	0.674
	Other	7	7.8%	NA		12.0	
	I	5	5.6%	26.0	0.257	24.0	0.763
Stage	II	75	83.3%	16.0		18.0	
	III	2	2.2%	NA		12.0	
	IV	8	8.9%	15.0		12.0	
Site	Head + Neck	45	50.0%	24.0	0.762	14.0	0.432
	Body + Tail	45	50.0%	31.0		18.0	
Differentiation	Well	11	12.2%	24.0	0.912	24.0	0.163
	Moderate	44	48.9%	18.0		18.0	
	Poor	35	38.9%	38.0		12.0	
*DDR* status	Other	53	76.8%	24.0	0.406	18.0	0.802
	Deleterious	16	23.2%	38.0		18.0	
*KRAS* status	Other	14	20.0%	24.0	0.965	14.0	0.307
	Deleterious	55	80.0%	31.0		18.0	
*TP53* status	Other	27	39.1%	NA	0.188	12.0	0.592
	Deleterious	42	60.9%	24.0		18.0	
*ARID1A* status	Other	60	87.0%	27.0	0.049	18.0	0.059
	Deleterious	9	13.0%	16.0		12.0	

Note

DDR: DNA Damage Repair; note that only 69 cases entered mutational analyses and 54 cases entered survival analyses.

### 3.2 Somatic mutations in pancreatic cancer

**FIGURE 1:**
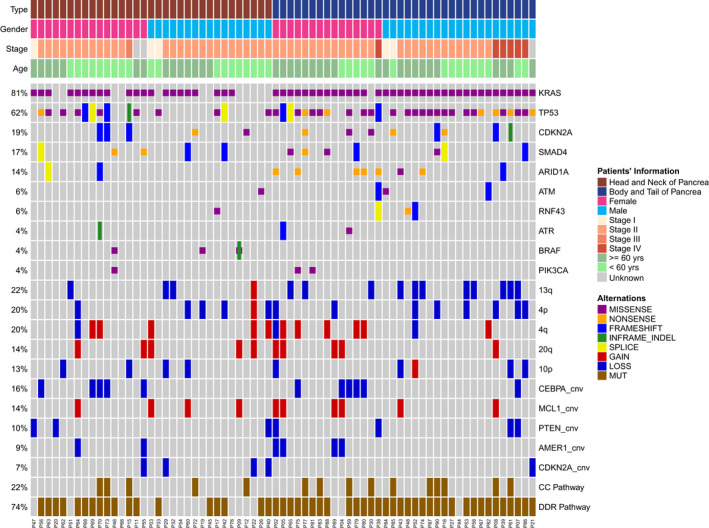
Distribution of genetic variations associated with pancreatic cancer. Distribution of individual gene Mutations (Top) and copy number variations (middle) in the cohort as assessed by target sequencing. Affected pathways in pancreatic cancer were shown on the bottom. Clinicopathological information was provided as bars on the top. Each column represents one patient

Sequencing was successful for specimens from 69 (76.7%) patients. Detailed genetic information for each patient was disclosed in Table S1. Frequently mutated genes included *KRAS*, *TP53*, *CDKN2A*, *SMAD4*, and *ARID1A*. The prevalence of mutational events in our cohort was 81% for *KRAS*, 62% for *TP53*, 19% for *CDKN2A*, 17% for *SMAD4*, and 14% for *ARID1A* (Figure 1, top bars). All *KRAS* mutations identified were missense mutations affecting hotspots. And all *TP53* mutations identified were predicted to be deleterious. For *CDKN2A*, all but one mutation were classified as oncogenic. The remaining mutation of unknown significance was an in‐frame deletion *CDKN2A* c.266_286del, which resulted in the deletion of codons 89 to 96, where no deleterious non‐truncating mutation was identified. Of note, the mutual exclusivity of mutations was insignificant in pancreatic cancer, except for *KRAS* and *BRAF* genes. The median tumor mutation burden was six for the cohort.

Copy number calling was successful in 69 (76.7%) cases. The cohort displayed gains of *MCL1* (14%, 10 out of 69), and losses of *CEBPA* (16%, 11 out of 69), *PTEN* (10%, 7 out of 69), *AMER1* (9%, 6 out of 69), and *CDKN2A* (7%, 5 out of 69) (Figure 1, lower bars). On chromosome level, pancreatic cancer displayed copy number changes of 13q, 4p, 4q, 20q, 10p. Interestingly, we identified 14% (10 out of 65) of patients who carried a chromosome 20q gain, which is frequently observed in pancreatic cancer in the western population^13^ (Figure 1, middle bars).

Pathway analyses revealed that the DNA damage repair (DDR) pathway was the most frequently altered pathway in pancreatic cancer, having excluded mutations curated in the Catalog of Somatic Mutations in Cancer (COSMIC). *AR1D1A* was the most significantly mutated gene in the DDR pathway. With 11 mutations distributing in 10 cases, it affected 14% of all valid cases. Twenty‐two percent of the patients had an altered cell cycling (CC) pathway (Figure 1, bottom bars), and *CDKN2A* was the most frequently mutated gene in the CC pathway, comprising 26% of all patients (Figure S2A).

### 3.3 Deleterious germline mutations in Chinese pancreatic cancer patients

Using corresponding normal tissue as control, we were able to distinguish between germline and somatic mutations. Of the 69 patients, 13 (14.44%) harbored a deleterious germline mutation. Table 2 detailed all patients with deleterious mutations. Among the 13 mutated genes identified, nine (69.23%) were associated with DNA damage response. Two *BRCA2* germline mutations, *BRCA2* p.F1460fs and *BRCA2* p.N2134fs, were detected in two patients, respectively. The patient with a *BRCA2* p.F1460fs mutation was a 47‐year‐old male. A 60‐year‐old female patient was found to carry a germline *BRCA1* p.R794X mutation. One 54‐year‐old male patient had a germline mutation in the *BRIP1* (BRCA1 interacting protein C‐terminal helicase 1) gene, which is known to increase the risk of ovarian, breast, and colon cancers.^14^ A deleterious germline mutation in the *PALB2* (Partner and Localizer of BRCA2) gene, which accounts for 3–4% of familial pancreatic cancer cases, was identified in a 53‐year‐old female patient.^15^ Other pathogenic germline mutations included mutations in *POLE* and *RAD51C*, which were also identified in different cohorts.^16^, ^17^ Germline mutations affecting other pathways included an *LZTR1* p.Y726X, a *PKHD1 c*.2592 + 1 G > T, and a recurrent *UGT1A1* p.Y486D mutations, which appeared in two patients.

**TABLE 2:** Deleterious germline mutations in pancreatic cancer patients

Patient ID	Sex	Age	Gene	AA change	Location
P02	Female	80	*LZTR1*	p.Y726X	BT^1^
P11	Female	66	*FANCC*	p.K369fs	HN^2^
P14	Male	47	*BRCA2*	p.F1460fs	HN
P16	Male	54	*BRIP1*	c.2492_2492+3delGGTA	BT
P17	Male	48	*PKHD1*	c.G2592+1T	HN
P29	Male	76	*UGT1A1*	p.Y486D	BT
P36	Male	59	*UGT1A1*	p.Y486D	BT
P37	Female	53	*PALB2*	p.S1169fs	HN
P45	Male	71	*FANCD2*	p.R794X	HN
P58	Male	59	*POLE*	c.A4729‐2G	BT
P59	Female	50	*RAD51C*	p.V169fs	BT
P77	Female	60	*BRCA1*	p.R794X	BT
P78	Female	70	*BRCA2*	p.N2134fs	HN

### 3.4 Actionable mutations in pancreatic cancer

Five cases carried actionable mutations. These included two cases carrying a germline *BRCA2* mutation each, one carrying a germline *BRCA1* mutation, one carrying a germline *PALB2* mutation, and one carrying a somatic *BRCA1* mutation. No *NTRK* fusion was identified. Mismatch repair genes were negative for all cases, except for a missense *MLH1* mutation of uncertain significance, which was in accordance with their microsatellite status.

Apart from the 51 *KRAS* hotspot mutations and 12 *CDKN2A* loss‐of‐function mutations, most potentially actionable mutations fell within the DDR and PI3K pathways.

Mutations associated with FDA‐approved therapies in other cancer types, which were potentially actionable, included three *PIK3CA* mutations (p.K111E, p.G118D, and p.E545K), two *BRAF* mutations (p.G466R and p.D594G, respectively), one *ERBB2* p.H878Y mutation, a MAP2K1 p.P124S mutation, and an *AKT1* mutation (p.E17K).

Fifteen potentially actionable somatic mutations in DDR genes were identified, including 10 *ARID1A* mutations, two *ATM* mutations, an *ATR* mutation, a *FANCI* mutation, and a *RECQL4* mutation. Of note, one patient harboring an *ARID1A* p.369fs mutation also had a concomitant *ARID1*A p.R1335X mutation and is, therefore, speculated to have bi‐allelic deactivation of the *ARID1A* gene. Two patients had concurrent *ARID1A/ATM* and *ARID1A/RECQL4* mutations, respectively. None of the patients had concurrent germline and somatic deleterious DDR pathway mutations.

### 3.5 The correlations of mutational signatures and tumor anatomic location in pancreatic cancer

**FIGURE 2:**
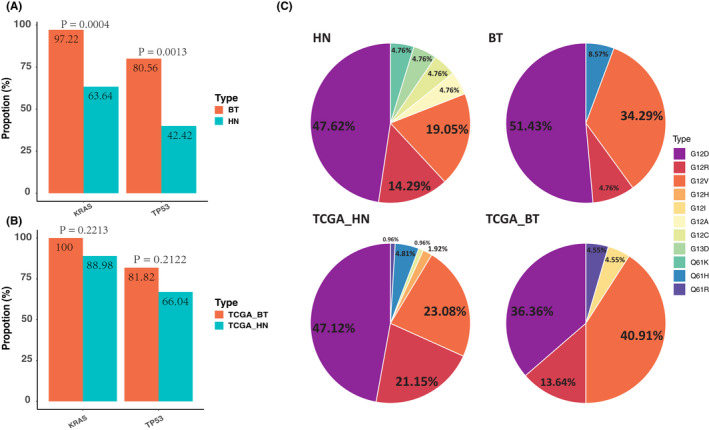
The correlation of mutational profile and tumor anatomic location in the pancreas. (A) Mutation frequency of KRAS and TP53 in tumors from HN and BT of pancreas based on our cohort and TCGA pancreatic cohort. (B) KRAS subtype distribution in tumors from HN and BT of pancreas based on our cohort and TCGA pancreatic cohort

To determine whether the mutational signature was correlated with tumor location, we assorted tumor samples into two groups: HN and BT tumors. As shown in Figure 2, the frequency of *KRAS* and *TP53* mutations showed a significant difference between HN and BT tumors. BT tumors had a higher ratio of mutations in *KRAS* and *TP53* than HN tumors (Figure 2A). The pancreatic cancer cohort from TCGA also showed an increased proportion of *KRAS* and *TP53* in the BT group compared to HN; however, without significant difference (Figure 2B).

Pancreatic tumors from HN and BT shared a similar KRAS subtype distribution pattern, predominately *KRAS* p.G12D, followed by *KRAS* p.G12V and *KRAS* p.G12R (Figure 2C, upper). This *KRAS* subtype distribution pattern was also observed in the TCGA cohort (Figure 2B, lower). On the contrary, HN tumors had a greater *KRAS* subtype diversity than BT tumors. *KRAS* subtype p.G12C, p.G12A, and p.G13D were observed in tumors from HN but not in tumors from BT (Figure 2B).

Regarding somatic mutations in the DDR pathway, a significantly higher rate in the BT tumors was identified (Figure S2B). When focusing on the ARID1A gene, although the rate of ARID1A mutation in BT tumors was three times that of the HN tumors, the difference was statistically insignificant. Differences in the pathological and molecular features of HN and BT tumors were detailed in Table 3.

**TABLE 3:** Clinicopathological and molecular characteristics of carcinomas arising from pancreatic head and neck or body and tail

Feature	Categories	Head and Neck		Body and tail		*P*
		Count	Column N%	Count	Column N%	
Age (years)	<=65	36	80.0%	31	68.9%	0.334
	>65	9	20.0%	14	31.1%	
Sex	Male	24	53.3%	25	55.6%	1.000
	Female	21	46.7%	20	44.4%	
Histological subtype	Ductal	42	93.3%	41	91.1%	1.000
	Other	3	6.7%	4	8.9%	
Stage	I	3	6.7%	2	4.4%	0.008
	II	41	91.1%	34	75.6%	
	III	1	2.2%	1	2.2%	
	IV	0	0.0%	8	17.8%	
Differentiation	Well	6	13.3%	5	11.1%	0.442
	Moderate	19	42.2%	25	55.6%	
	Poor	20	44.4%	15	33.3%	
*DDR* status	Other	29	87.9%	24	66.7%	0.048
	Deleterious	4	12.1%	12	33.3%	
*KRAS* status	Other	12	36.4%	2	5.6%	0.002
	Deleterious	21	63.6%	34	94.4%	
*TP53* status	Other	19	57.6%	8	22.2%	0.006
	Deleterious	14	31.1%	28	77.8%	
*ARID1A* status	Other	31	93.9%	39	80.6%	0.154
	Deleterious	2	6.1%	7	19.4%	

### 3.6 Survival analysis

**FIGURE 3:**
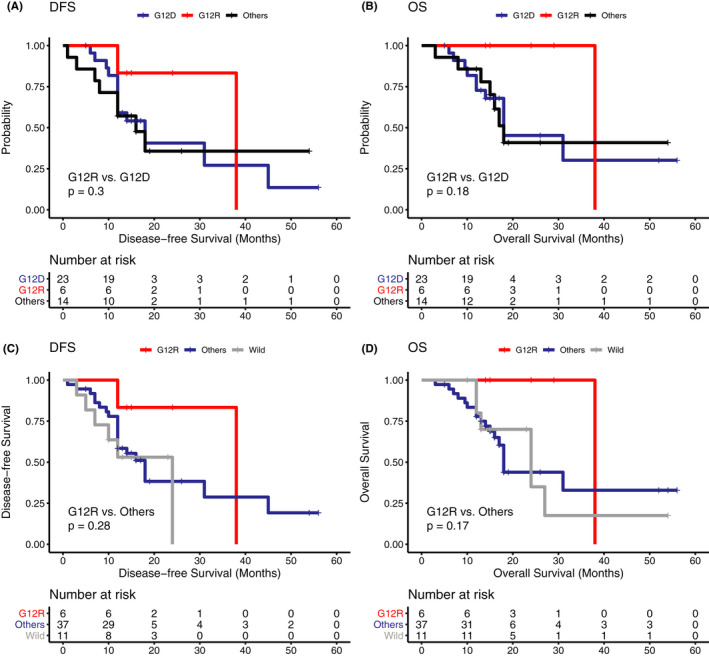
The correlation of KRAS subtype with disease‐free survival and overall survival in pancreatic cancer. Comparison of KRAS p.G12D and p.G12R in disease‐free survival (A) and overall survival (B). Comparison of KRAS WT, p.G12R, and other KRAS subtypes in disease‐free survival (C) and overall survival (D)

On Kaplan–Meier analysis, *KRAS* subtypes were not associated with significant differences in DFS and OS. Compared to cases with a *KRAS* p.G12D mutation, those carrying a *KRAS* p.G12R mutation had a better median DFS and OS, which both were 38 months (Figure 3A&B). Meanwhile, patients with a *KRAS* p.G12R mutation also showed better DFS and OS than patients with a wild‐type *KRAS* gene or other *KRAS* subtypes (Figure 3C&D).

**FIGURE 4:**
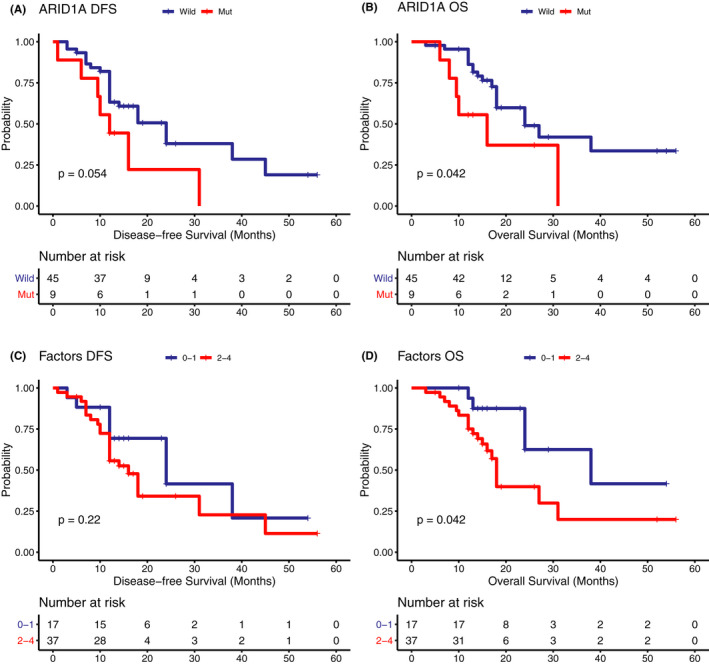
Mutated ARID1A gene alone and the number of mutated driver genes were associated with more unsatisfactory disease outcome. (A) Disease‐free survival and (B) Overall survival of patients according to the mutated ARID1A gene. (C) Disease‐free survival and (D) Overall survival of patients according to the number of mutated driver genes

It has been reported that pancreatic cancer patients with mutated driver genes suffered from a poorer OS.^18^ In our cohort, each driver gene mutation alone, or combined, including *KRAS*, *CDKN2A*, *TP53*, and *SMAD4*, were not associated with DFS or OS (Data not shown). Interestingly, mutations in *ARID1A*, a candidate driver gene in pancreatic carcinogenesis, were associated with inferior DFS and OS. Patients with mutated *ARID1A* showed a median DFS and OS of 12 and 16 months, respectively, while those with a wild‐type *ARID1A* had a median DFS and OS of 24 months (Figure 4A&B). However, no significant difference in DFS and OS was observed in the TCGA patient cohort (Figure S3A&B).

Then, we investigated the number of mutated genes among driver genes (*KRAS*, *CDKN2A*, *TP53*, and *ARID1A*). The OS of patients with 0–1 mutated driver genes was significantly longer than that of patients with 2–4 mutated driver genes (median OS, 38.0 vs. 18.0 months). No significant difference was found in DFS between these two groups (Figure 3C&D). On the contrary, in the TCGA pancreatic cohort, we observed a significant difference in DFS but not in OS between patients with 0–1 mutated 2–4 mutated driver genes (Figure S3C&D).

The impact of three (4.35%) macroscopic residual tumors on DFS and OS was also evaluated by Kaplan–Meier Test. While these cases did demonstrate inferior DFS and OS, the statistics were insignificant.

Regretfully, none of the above factors were significantly associated with OS or DFS in multivariate analyses (Table S2).

## 4 DISCUSSION

In this study, we investigated the clinicopathological characteristics and mutation profile of 90 Chinese pancreatic cancers. While consensus targeting therapeutic options is only available for a small fraction of the cases based on current evidence, our data demonstrated that pancreatic cancer had a broad spectrum of potentially actionable alterations.

In this cohort, we learned that 83% of the cases carried a *KRAS* mutation, which was relatively lower than the Western population, as reported in TCGA (90%‐96%). In a preprint paper under consideration in Cancer Cell International (https://www.researchsquare.com/article/rs‐60530/v1), which adopted a Chinese PDAC cohort consisted of 195 patients. A *KRA*S mutation was found in 83.6% of their cases. No significant difference in the prevalence of *KRAS* mutations was found in these two cohorts. Therefore, it is reasonable to assume that the relatively low *KRAS* mutation rate might have reflected difference between races. *KRAS* activating mutations used to be considered undruggable, probably because of the lack of “pouch” formation in the variants, making it difficult to bind to. Strategies to target a *KRAS* mutation is either by covalent inhibition or inhibiting its downstream signals. Both yielded promising results. Covalent inhibitors are difficult to find and require different agents for different variants.^19^ Currently, only *KRAS* p.G12C has been successfully targeted. However, *KRAS* p.G12C mutation only occurred in a small fraction of pancreatic cancer, as evident in our cohort and the TCGA cohorts. More patients may be immediately benefited from inhibiting the downstream elements, such as MEK. Recent studies have partially revealed the underlying mechanism of rapid resistance acquisition to MEK inhibitors, which was associated with autophagy dependency. The addition of chloroquine, capable of inhibiting autophagy in this context, shed light on the solution to resistance development.^20^ In previous retrospective studies, *KRAS* p.G12D was found a poor prognostic factor for OS across different cancer types.^21^, ^22^ In our cohort, *KRAS* p.G12D carriers showed shorter DFS and OS than those with a p.G12R, which were not different from the previous reports.

Most genes with a germline mutation detected in our cohort belonged to the DDR pathway. The prevalence of *BRCA2* mutation was 2.90% (2 out of 69) in our cohort, assimilating the study results in the western population (1.95%, 59 out of 3060).^23^ The prevalence of other altered DDR genes, including *BRCA1*, *BRIP1*, *RAD51C*, and *FANCC*, were slightly higher than in the literature, which was probably incidental in the context of a relatively small denominator. The similarities in the profile of germline DDR gene mutations suggest the generalizability of relating studies in the western populations to Chinese patients.

Recently, Poly (ADP‐ribose) polymerase (PARP) inhibitors have emerged as a treatment option for solid tumors with DDR gene deficiencies. Olaparib, a PARP inhibitor, has been approved by the FDA in pancreatic cancer patients with *BRAC1/2* or *PALB2* mutations.^24^, ^25^ In the present study, while only four patients had a germline *BRCA1/2* or *PALB2* mutation, another 7.24% (5 out of 69) patients had a deleterious germline mutation in other DDR genes, and 22 (31.88%) cases had at least one deleterious DDR gene mutation, germline, and somatic combined. Our data suggested the urgent need to extend PARP inhibitor trials to include patients with other germline and somatic DDR gene mutations.

Compared to TCGA data, this Chinese cohort exhibited relatively lower *SMAD4* frequency yet higher *ARID1A* frequency, suggesting *ARID1A* may be a more potent driver in Chinese than *SMAD4*. *ARID1A* gene has been reported to participate in many aspects of carcinogenesis. It is involved in DNA damage response and has been demonstrated to confer sensitivity to combined radiotherapy and PARP inhibitor therapy.^26^ It is also considered to have a role in epigenetic modulation in tumor biology, as evidenced by the synthetic lethality of EZH2 inhibition in *ARID1A*‐mutated tumors.^27^ Furthermore, *ARID1A* deficiency sensitized PC to PI3K/AKT inhibition in vitro.^28^ In the present study, we reported a statistically insignificant yet clinically important predilection of *ARID1A* somatic mutations in BT tumors. With 19.4% (7 out of 36) BT cases having at least one deleterious somatic *ARID1A* mutation, our data provided essential information to facilitate clinical trial allocation that aims at this very promising target. Altered *ARID1A* was also found to be associated with significantly shorter DFS and OS in the cohort, in concordance with the result of an earlier study enrolling 109 micro‐dissected pancreatic ductal adenocarcinoma cases.^29^ Worse survival was also reported in patients with *ARID1A* gene mutations in a cohort of 22 PDAC patients treated with neoadjuvant chemoradiation therapy.^30^ This study also analyzed the prognostic value of *ARID1A* mutations and *KRAS* p.G12R mutation, along with other clinicopathological factors (Table S2). In univariate analysis, *ARID1A* mutations tend to be associated with DFS and OS. However, the association became insignificant in the subsequent multivariate analysis. The result might be due to the presence of confounding factors and small sample size.

The present study has limitations. The sample size is limited compared to existing studies, although a relatively small sample size had enabled the possibility of analyzing the mutation profile case by case. Also, to ensure the availability of sufficient tissue and intact clinicopathological information for subsequent analyses, we have discarded quite a few cases in the library; thereby, the cohort is neither randomized nor consecutive. However, the point of this preliminary report has been that the druggability of PC should never be overlooked.

In summary, in the present study, we confirmed the importance of sequencing tumor‐associated genes in pancreatic cancer to inform therapeutic options and clinical trial allocation. The relationship between mutational features and pancreatic tumor locations was established. Also, we showed the correlation of the altered driver gene *ARID1A* and *KRAS* subtype with DFS and overall OS.

In conclusion, pancreatic tumors with different anatomic locations showed a difference in mutation profiles, which could divert future treatment options for HN and BT tumor patients.

## CONFLICTS OF INTEREST

Authors RL and RL were employed by the company Geneseeq Technology Inc. Author YS was employed by the company Nanjing Geneseeq Technology Inc. The remaining authors declare that the research was conducted in the absence of any commercial or financial relationships that could be construed as a potential conflict of interest. The funders had no role in the design of the study; in the collection, analyses, or interpretation of data; in the writing of the manuscript, or in the decision to publish the results.

## AUTHOR CONTRIBUTIONS

JL analyzed the data and developed the introduction and discussion section of the manuscript. RL and RY analyzed and visualized the data and develop the method and result section of the manuscript. XL and ZZ followed the patients. JS and HW revised the manuscript; JG provided clinical data of the patients. YS provided resources for the sequencing of the specimens. ZL supervised the study and revised the manuscript.

## Supporting information

Table S1

Table S2

Supplementary Material

## Data Availability

The data that support the findings of this study are available from the corresponding author upon reasonable request.
