# Supplementary material for: Genetic aberrations in Chinese pancreatic cancer patients and their association with anatomic location and disease outcomes
Source: Cancer Med. 2020 Dec 22;10(3):933–43. doi: 10.1002/cam4.3679 (PMC7897942; doi:10.1002/cam4.3679)
Supplement: Supplementary file 2 — Table S2 [file CAM4-10-933-s002.docx]

Supplementary Table 2. Univariate and multivariate analysis of the prognostic value of *KRAS* G12R and *ARID1A* according to sex, age and stage. HR: hazard ratio. Inf: infinite. *: P＜0.05.

| **Factors** | **Univariate analysis** | | | | | | | |  |  |  |  |  |
| --- | --- | --- | --- | --- | --- | --- | --- | --- | --- | --- | --- | --- | --- |
|  | **DFS-HR (95%Cl)** | | **DFS-*P*** | | | **OS-HR (95%Cl)** | | **OS-*P*** | | |  |  |  |
| **Sex** | 0.82(0.39~1.73) | | 0.62 | | | 0.91(0.40~2.08) | | 0.84 | | |  |  |  |
| **Age** | 0.98(0.95~1.02) | | 0.53 | | | 0.99(0.95~1.03) | | 0.69 | | |  |  |  |
| **Stage** | 2.11(0.79~5.63) | | 0.13 | | | 2.91(1.06~7.98) | | 0.03 * | | |  |  |  |
| **KRAS G12R vs G12V/D** | 2.28(0.52~9.90) | | 0.26 | | | 4.29(0.56~32.58) | | 0.15 | | |  |  |  |
| **ARID1A WT vs**  **Mut** | 2.17(0.92~5.14) | | 0.07 | | | 2.47(0.96~6.30) | | 0.05 | | |  |  |  |
| **Factors** | | **Multivariate analysis** | | | | | | | | | |  | |
|  |  | **DFS-HR (95%Cl)** | | **DFS-*P*** | **OS-HR (95%Cl)** | | **OS-*P*** | | | | | |  |
| **Sex** | | 1.16(0.40~3.36) | | 0.78 | 1.34(0.42~4.29) | | 0.61 | | | | | |  |
| **Age** | | 0.99(0.95~1.04) | | 0.92 | 0.99(0.93~1.04) | | 0.76 | | | | | |  |
| **Stage** | | 2.46(0.76~7.92) | | 0.13 | 2.99(0.90~9.95) | | 0.07 | | | | | |  |
| **KRAS G12R vs G12V/D** | | 1.95(0.37~10.35) | | 0.42 | 3.24(0.34~30.16) | | 0.30 | | | | | |  |
| **Sex** | | 1.16(0.51~2.61) | | 0.71 | 1.55(0.58~4.18) | | 0.37 | | | | | |  |
| **Age** | | 0.97(0.93~1.01) | | 0.19 | 0.98(0.94~1.02) | | 0.39 | | | | | |  |
| **Stage** | | 1.55(0.49~4.91) | | 0.44 | 2.34(0.72~7.59) | | 0.15 | | | | | |  |
| **ARID1A WT vs**  **Mut** | | 2.51(0.81~7.73) | | 0.10 | 2.51(0.72~8.78) | | 0.14 | | | | | |  |
