# Supplementary material for: Genetic aberrations in Chinese pancreatic cancer patients and their association with anatomic location and disease outcomes
Source: Cancer Med. 2020 Dec 22;10(3):933–43. doi: 10.1002/cam4.3679 (PMC7897942; doi:10.1002/cam4.3679)
Supplement: Supplementary file 3 — Supplementary Material [file CAM4-10-933-s003.docx]

Supplementary Figure 1-3

**Supplementary Figure1. Flow chart of patient criteria**


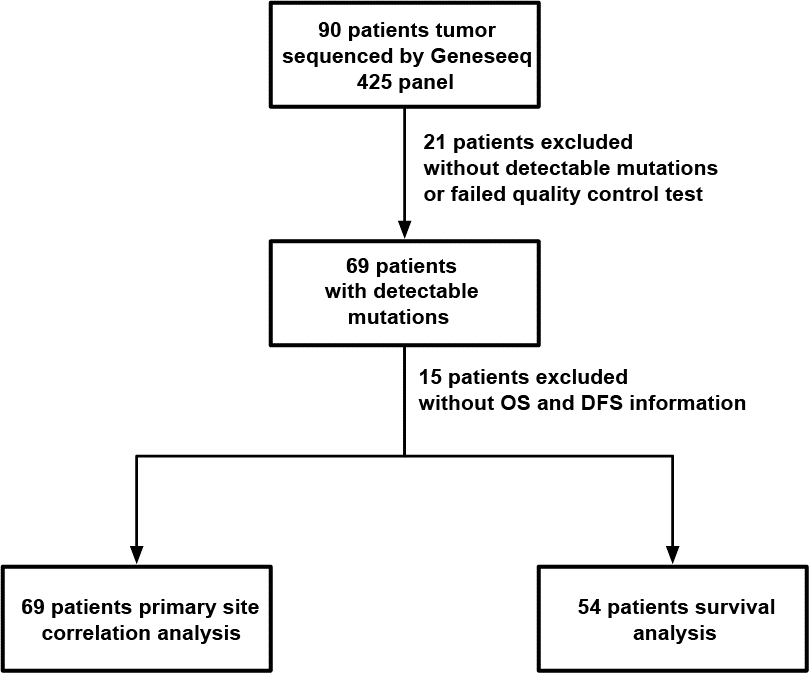


**Supplementary Figure 2. Altered pathways in pancreatic cancers.** (A) Distribution of mutated genes in cell cycle and DNA damage repair pathways was shown. Each column represents one patient.(B) Distribution of germline and somatic mutations from DNA damage repair pathway in HN-originated and BT-originated pancreatic tumors.


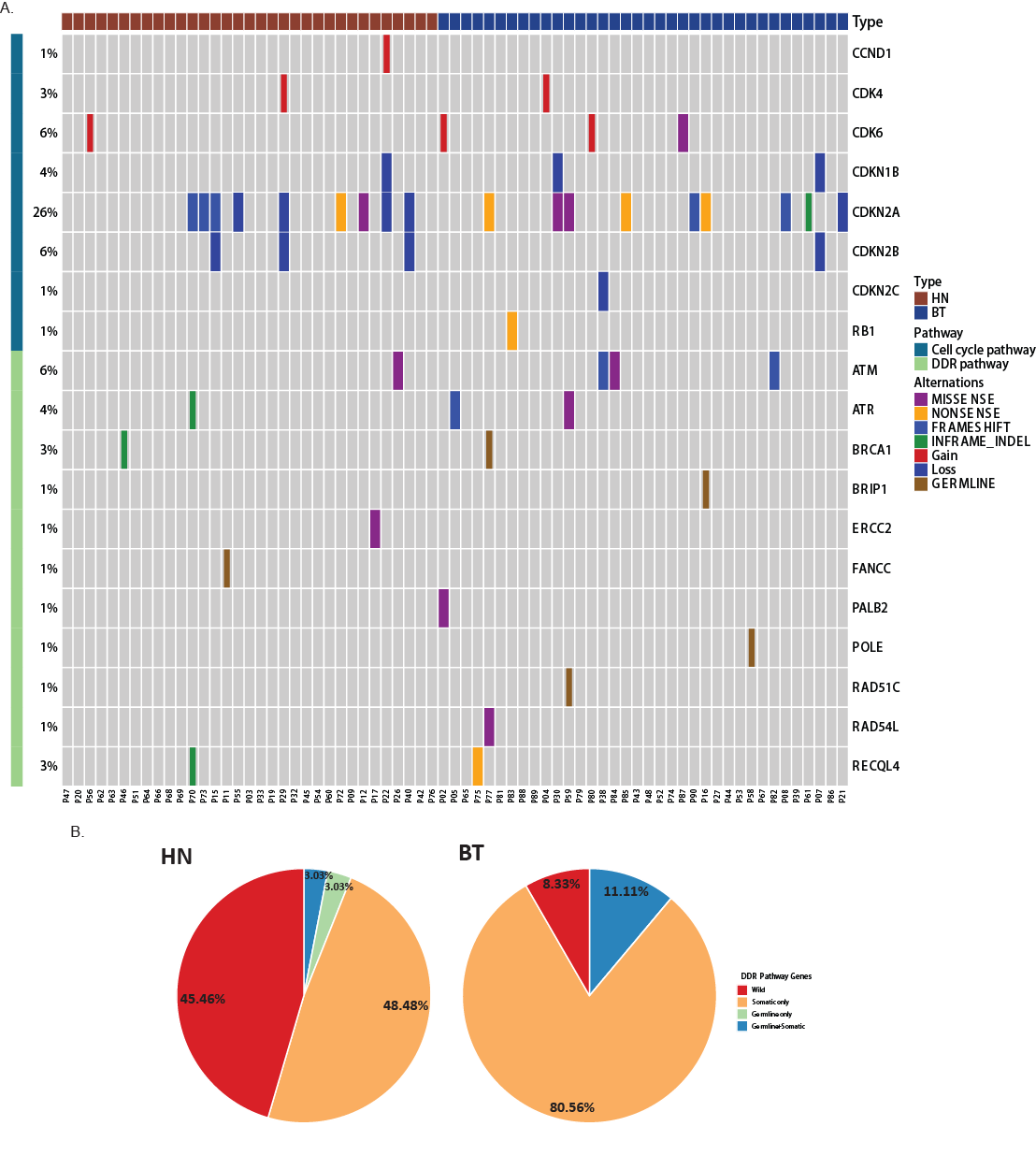


BT: body and tail of pancreas; HN: head and neck of pancreas;

CC: cell cycle; DDR: DNA damage repair

**Supplementary Figure3. The correlation of mutated *ARID1A* gene alone and mutated driver genes with disease-free survival and overall survival in TCGA pancreatic cohort.** (A) Disease-free survival and (B) Overall survival of patients according to mutated *ARID1A* gene. (C) Disease-free survival and (D) Overall survival of patients according to the number of mutated driver genes.


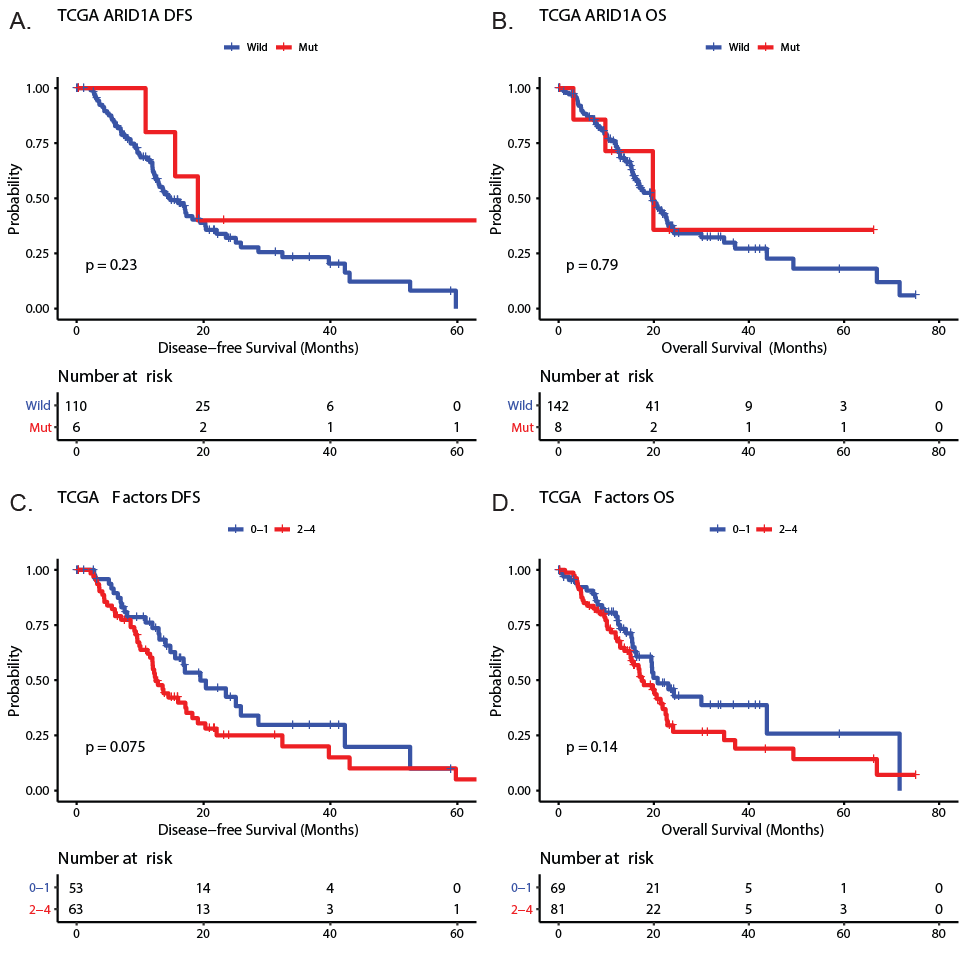


Wild: patients with wild type *ARID1A* gene; Mut: patients with mutated *ARID1A* gene;

0-1:0-1 mutated driver genes; 2-4:2-4 mutated driver genes
